# Threats to the Reintroduction Program of the Northern Bald Ibis (*Geronticus eremita*) in Italy: A Forensic Investigation

**DOI:** 10.3390/ani13010066

**Published:** 2022-12-23

**Authors:** Francesca Maccagnan, Lorena Di Benedetto, Giulia Rosa, Rosario Fico

**Affiliations:** Centro di Referenza Nazionale per la Medicina Forense Veterinaria, Istituto Zooprofilattico Sperimentale del Lazio e della Toscana “M. Aleandri”, 58100 Grosseto, Italy

**Keywords:** forensic veterinary medicine, illegal hunting, reintroduction program, endangered species, poaching, *Geronticus eremita*, Northern Bald Ibis, wildlife

## Abstract

**Simple Summary:**

The Northern Bald Ibis is one of the world’s rarest birds, and it was reintroduced in Europe. Its survival is strongly threatened by illegal hunting in Italy. This study used a forensic approach to analyze all of the cadavers found in Italy between 2016 and 2022, which were suspected to have been victims of illegal hunting. In our study, illegal hunting was the major cause of death in this species, mainly during the hunting season. Natural causes of death accounted for the minor part, confirming the importance of approaching these cases with a forensic method. Forensic veterinary medicine is a science that tries to reach not only to obtain a diagnosis but also evidence (when, where, how, and (hopefully) who) and it must be used, together with other forensic sciences, to stop poaching, which is a serious problem that is leading many species to extinction.

**Abstract:**

The Northern Bald Ibis *Geronticus eremita* is an endangered species, and recently it was reintroduced in Europe by the Waldrappteam. The reintroduction program has been strongly threatened by several sudden deaths, mainly in Italy. The present study used a forensic approach to analyze all of the 27 Northern Bald Ibises found dead in Italy between 2016 and 2022, which were suspected to have been victims of poaching, and it followed the veterinary forensic guidelines. Human-related causes accounted for 60% of the deaths, including illegal hunting (30%), blunt force trauma (26%), and electrocution (4%). Natural causes, starvation (15%), predation (11%) and disease (7%), accounted for 33% of the deaths. 7% of the causes of deaths remained undetermined. This study uses a forensic approach to analyze, for the first time, the main causes of death in *Geronticus eremita* and highlights the relevance of detecting illegal actions related to endangered species and stopping the phenomenon of poaching.

## 1. Introduction

The Northern Bald Ibis (*Geronticus eremita)* is one of the rarest birds in the world, and recently it was reintroduced in Europe through a LIFE+ project “Reason for hope” by the Waldrappteam. This animal was once widespread throughout the Middle East, northern Africa, and southern and central Europe. This species disappeared from Europe over 400 years ago, but the breeding population persisted in Morocco, Algeria, Turkey, and Syria. The main population occurs in Morocco and numbers just over 100 breeding pairs [1,2].

In 2002, the project Waldrappteam initiated a 12-year feasibility study to develop methods for the establishment of migratory Northern Bald Ibis colonies. Spring migration starts in late March, and autumn migration starts at the beginning of August, but birds regularly remain north of the Alps up to mid-September. The migration routes cross the Alps to a wintering area in southern Tuscany (Italy). A major challenge for the project is losses caused by illegal hunting during the autumn migration, mainly in Italy, where the animals’ wintering site and the major part of their migratory flyway are located [2,3]. Illegal hunting, also known as poaching, has a strong demographic and economic impact on this project. Poaching is a worldwide problem that can affect species through large or small-scale changes in species’ distribution or shifts in species’ behaviour. In addition, species can indirectly be affected by poaching through changes in species composition within a protected area or a reduction in resources due to altered ecosystem structure or function [4].

Since the end of 2016 the Centro di Referenza Nazionale per la Medicina Forense Veterinaria (CeMedForVet) has been involved in the fight against the poaching of this species. Furthermore, all of the animals were provided with GPS, which allowed the position and the activity of every bird to be detected at least once a day, in order to gather more possible information in case the animals suddenly die and to distinguish between human-related and natural causes of death. As a matter of fact, a forensic approach contributes to justice, providing the authorities with evidence, such as autopsy findings and diagnosis; for this reason, it is crucial in fighting against poaching [5]. Several studies have investigated the causes of death in endangered species, in particular human-related causes, which mainly threaten migrant birds; however, very few studies have taken advantage of the forensic approach [6,7,8]. In particular, the Northern Bald Ibis is one of the 20 most threatened species by illegal hunting [9], although no forensic studies on this species exist in the literature. Given the need to reduce poaching [4], the aim of this study is to provide the stakeholder with information on the Northern Bald Ibis’ major causes of death and to highlight the positive implication and contribution of a forensic study in a conservation program. Indeed, human-related causes of death are still the major issue in the survival of this species.

## 2. Materials and Methods

27 Northern Bald Ibises found dead in Italy between 2016 and 2022, likely due to illegal hunting, were collected by the judicial authorities and delivered to the CeMedForVet at Istituto Zooprofilattico Sperimentale del Lazio e della Toscana (IZSLT), in Grosseto, 58,100 Italy. In total, 2 of these cases, which were suspected to have been victims of illegal hunting, were analyzed in other diagnostic laboratories in Italy and subsequently delivered to our center to confirm the cause of death. The Waldrappteam provided all of the animals with GPS, which allowed for the position and the activity, and especially the movements before the death of every bird, to be detected at least once a day. The GPS provides forensic veterinarians with crucial data that may help them to better understand the dynamic of death. Therefore, historical data, including the GPS information, the animal’s location and activity, and the clinical history, if available, were collected by the authorities and the Waldrappteam volunteers and transmitted to the pathologists. The Northern Bald Ibises were analyzed by CeMedForvet following forensic veterinary guidelines and using a forensic approach, making use of all of the additional available data [10]. After the evaluation of the available data, when deemed necessary by the authorities or forensic veterinarians, some animals were radiographed and/or analyzed by a ballistics expert.

Upon delivery, the animals, identified with bird rings, were registered with an identification number and then stored at −20 °C. In order to avoid tissue and organ alterations or modifications, the autopsies were performed after defrosting the animals at refrigeration temperature (0–4 °C). The autopsies were performed by two forensic veterinarians, one of whom was in charge of the autopsy, while the other was in charge of taking the photographs. This method was essential to prevent the veterinarian in charge of the “clean actions” such as taking pictures or writing from coming into contact with the animals. Moreover, in this way the veterinarian in charge of the photographs could carefully document the autopsy, providing important evidence. Prior to the autopsy, the forensic veterinarian gathered all of the useful information for the investigation. When illegal hunting was suspected, the radiographic analysis or other enquiries deemed necessary were performed before the autopsy, whereas in the case of this suspicion arising during the autopsy, they were only performed afterwards. When radiographs were requested, due to the need for obtaining clear images without overlapping, the dead animals were conveyed to the clinic frozen with their wings open and their heads bent to the right, and a total body X-ray analysis was executed to include all of the animals’ parts.

All of the autopsies were performed in the autopsy room of the IZSLT, following the veterinary forensic guidelines [10]. If the autolysis did not preclude such tests, postmortem examinations included a detailed evaluation of the body, the organs, and the lesions. Furthermore, by analyzing the pectoral muscles and the general muscle status, the nutritional status was established. An animal with extremely low muscle mass, low muscle mass, or good muscle mass was defined having a ‘poor’, ‘fair’, or ‘good’ nutritional status, respectively.

Moreover, the palpation of the animal was carefully executed in order to detect possible fractures. During the autopsy, the skin and subcutis were the first elements analyzed to detect traumatic lesions or other abnormalities. The animals were skinned and the whole subcutaneous tissue was ispectioned because many cutaneous lesions could have been undetectable due to the plumage or the conservation status of the animals.

In the case of shot animals, the trajectory of the metal findings was analyzed from the point of entrance and from the resulting injuries, to the internal organ lesions or to the point of exit. Moreover, by analyzing the body and the organ injuries of a hunted bird, together with the GPS data and the anamnesis, we tried to devine the dynamic of the event, for instance, if the animal was in flight or if it was on the ground when shot, and the direction of the shot. Furthermore, by measuring the distance between the two furthest holes, a ballistics expert was able to understand the distance between the animal and the point of shot [11].

When injuries were attributable to bite wounds intra vitam, we concluded that the cause of death was predation. Then, the bite marks were measured and carefully analyzed to try to identify the species of predator [12,13,14].

At autopsy, tissue samples were collected and submitted to appropriate laboratories for virologic, parasitologic, and toxicologic analysis based on lesions and differential diagnoses, and these varied case by case. In general, in the case of suspected poisoning, gastric contents and liver samples were analyzed by the IZSLT toxicological laboratory in Florence, using the GC-MS method and following the GTFI guidelines [15]. A kidney sample was analyzed by the IZSLT histological laboratory in Rome, Italy. The necessary parasitological and virological examinations were performed by the IZSLT parasitological and virological laboratory in Grosseto and Rome, Italy, respectively.

The causes of death were categorized as “illegal hunting” when the animal was killed by firearms; “trauma” when evident blunt-force traumatic lesions were detected and the available data confirmed the suspicion; “predation” when clear intra vitam bite injuries were found; “electrocution” when burn marks and absence of evidence of other causes of death were identified; “disease” if specific pathologies or pathogens were diagnosed; “starvation” if the animal was extremely emaciated, no pathologies were found, and the available data confirmed the suspicion; and “undetermined” if no diagnosis was reached.

## 3. Results

**Figure 1:**
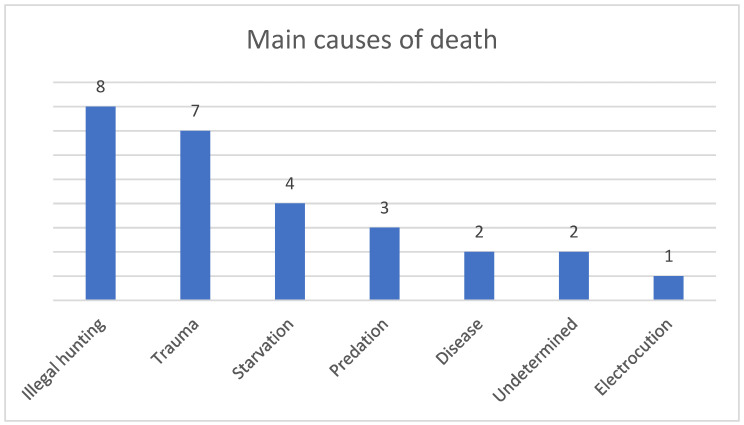
Main causes of death in reintroduced Northern Bald Ibis analyzed at CeMedForVet in the period 2016–2022.

During the study period (2016–2022), 27 Northern Bald Ibis were analyzed at the CeMedForVet. The cause of death was determined for 25 of the 27 (93%) birds necropsied. Causes of death (Figure 1) include predation (*n* = 3, 11%), trauma (*n* = 7, 26%), disease (*n* = 2, 7%), starvation (*n* = 4, 15%), illegal hunting (*n* = 8, 30%), electrocution (*n* = 1, 4%), and undetermined (*n* = 2, 7%). Individual causes of death are presented in Table 1. Post mortem decomposition and the necropsies performed in other diagnostic laboratories on the cadaver were confounding factors in the determination of the cause of death. Human-related causes of death (illegal hunting, trauma, and electrocution) were more frequent (*n* = 16, 60%) than natural causes of death (disease, predation, and starvation) (*n* = 9, 33%). Starvation cases were attributed to natural causes since no pathologies were found and starvation is caused by multiple factors, such as food availability, seasonal temperatures, and predation pressure [16]. Therefore, human-related causes were excluded.

In cases of predation, the distance between the lesions in the cutis and subcutis of the predated animals measures between 1.5 and 2.5 cm, which is consistent with a small carnivore.

All of the animals found dead were collected in Northern and Central Italy (Table 1), notably in Tuscany (*n* = 20, 74%), Veneto (*n* = 3, 11%), and Lombardy (*n* = 2, 7%). Only one animal was found in Liguria (4%) and one in Emilia Romagna (4%). Most dead Bald Ibises were found during autumn and winter between September and February (*n* = 18, 67%), while in spring and summer (March–August) only 9 animals (33%) were found. Moreover, nearly all of the shot animals (*n* = 7, 87%) were found dead during the hunting season (September–January).

The diagnostic tests (Table 2) showed that all of the tested animals (*n* = 9) were negative in the toxicological examination and in the virological exam (*n* = 6). Moreover, three animals underwent parasitological examination, and two of them (67%) tested positive, one for ascaridiosis and the other one for strongyloides. In 14 cases, forensic veterinarians requested a radiography or ballistics examination, 13 animals were radiographed, and 2 underwent ballistics analysis. In 8 (57%) cases, the radiography showed metallic radipacity compatible with ballistics elements. The histological examination was performed in seven cases to investigate other possible causes of death.

## 4. Discussion

This study highlights the main causes of death in the Northern Bald Ibis population in Italy in order to make a contribution to the reintroduction and conservation of this endangered species. All of the cases were analyzed in accordance with the veterinary forensic guidelines [10], together with further specialists, such as ballistics experts.

Italy is one of the three Mediterranean countries with the highest numbers of birds illegally killed, with an average of 5,400,000 individuals per year. In total, 10 of the 20 bird species with the largest estimated number of individual birds illegally killed per year in the Mediterranean are killed in Italy. In addition, the Northern Bald Ibis, together with other 7 migrant species in Italy, is one of the 20 endangered species threatened by illegal hunting, with the highest ratio between the estimated number of individuals killed and the European population size [9]. A major challenge for the Northern Bald Ibis reintroduction project is losses caused by illegal hunting during the autumn migration. Therefore, 31% of the Ibises in Italy die due to illegal hunting. During the feasibility study of the Ibis reintroduction project (2002–2013), up to 71% of the losses were attributable to illegal hunting in Italy that decreased since 2014 [2,17]. The Waldrappteam annual reports show an increase in illegal hunting (from 12% in 2016 to 28% in 2020, and 19% in 2021, with an average of 14%) [18,19,20,21,22,23].

In this study, the primary cause of death in the Northern Bald Ibis was illegal hunting. Our data are slightly non-linear with those in the Waldrappteam reports because they show a higher incidence of illegal hunting. Since we only received animals suspected to have been illegally hunted, our sample might have overestimated the incidence of poaching. However, all of the deaths due to illegal hunting occurred along the Italian flyway and during autumn migration (when hunting is permitted according to current regulations (Law 157/92)). Moreover, in Italy there are an estimated 1,222,537 valid firearms licenses, of which 631,304 are for hunting uses [24]. Considering these data and the fact that the migrating Ibises died due to illegal hunting almost only during the autumn migration, coinciding with the hunting season, we suggest that the poaching problem may be related to people holding a legal hunting permit. The threat of illegal hunting was the main reason why the whole Northern Bald Ibis population was fitted with GPS tags, which allowed the position and the activity of every bird to be detected at least once a day [25]. This measure, together with an education program for hunters, may be a relevant measure to stop poaching. As a matter of fact, the GPS data, together with a forensic approach, are extremely useful in understanding the cause of death and in distinguishing between human and natural causes. Wildlife animals have no anamnesis, and their traumatic injuries are often nonspecific. Indeed, injuries such as traumatic impact lesions on the wildlife animals are recognizable only with a forensic approach. For this reason, the GPS data and all of the further information given to the pathologists are crucial in solving the case.

In our study, trauma was a significant cause of death in this population. However, only three birds had a confirmed cause of predation, and only one bird had confirmed lesions due to electrocution. Therefore, seven cases showed blunt force traumatic injuries, which were attributable to human-related causes, such as collision trauma caused by motor vehicles. It was possible to reach this diagnosis through a forensic approach, using the GPS data.

Our data regarding traumatic lesions are consistent with the Waldrappteam annual reports, apart from electrocution, which is more frequent in the reports [18,19,20,21,22,23]. This may be due to the easier recognition of the electrocution lesions and the consequent exclusion of the poaching suspicion, that is, the necessary prerequisite to deliver us the cadaver. Indeed, predation and electrocution gross lesions are easier to recognize. Electrocuted birds often die immediately and are found near a power pole or beneath a power line. Moreover, the hallmark of electrocution is burn marks and an absence of evidence of other causes of death [26]. On the other hand, lesions attributable to predation are clearly evident during the inspection of the cutis and the subcutaneous tissue, and by measuring the distance between the cutaneous wounds left by the canine teeth it is also possible to recognize the size of the predator [12,13,14,27]. In our study, in these three specific cases predation was carried out by a small carnivore, most likely a Red Fox (*Vulpes vulpes*).

These data are consistent with the Waldrappteam annual reports. Indeed, between 2016 and 2022 the animals that died due to disease averaged 13% [18,19,20,21,22,23]. However, it must be highlighted that we received animals from the authorities and from the volunteers that had forensic suspicions, and for these reasons our data could have underestimated the extent of the problem.

The cause of death for two birds remains undetermined, due to the advanced decomposition that limited further examination. Moreover, the two animals were previously sectioned in another diagnostic laboratory compromising the corpses, and no anamnesis of the animals was delivered to the pathologists.

The data of our study are consistent with the literature. Hunting and overexploitation are major causes of species extinctions in birds and mammals [28]. As a matter of fact, human-induced extinction rates are significantly higher than natural background rate and are predicted to increase [29]. In total, 38% of the bird species in the world are actually threatened by illegal hunting [6], and in the Mediterranean countries 11–36 million individuals per year are estimated to be illegally killed, many of them migrating [9]. Indeed, the results of our study suggest that human-related causes of death are highly prevalent, about twice as high as natural causes, in Northern Bald Ibises in Italy. However, human actions are a crucial issue in the conservation of endangered species throughout the world.

## 5. Conclusions

The reintroduction program of the Northern Bald Ibis in Europe has been severely threatened by illegal hunting, especially in Italy. The persistence of this phenomenon throughout the years highlights the importance of performing a forensic, holistic investigation in order to protect endangered species. In total, 87% of the Ibises that died as a result of illegal hunting were found during the hunting season. Consequently, it is plausible that the poachers who caused these deaths are regular hunting license holders. Therefore, these data provide the stakeholders with a new focus and a common purpose. We deeply believe that a forensic approach, together with social sensibilization, may reduce the human impact that is threatening the Northern Bald Ibis and all of the endangered bird species in the world.

## Figures and Tables

**Table 1:** Causes of death and key information related to reintroduced Northern Bald Ibis analyzed at CeMedForVet in the period 2016–2022.

Bird Identification	Date of Death	Location of Death (Italy)	Postmortem Condition	Nutritional Status	Cause of Death	Diagnostic Tests Performed	Additional Details
xxxx675	October 2016	Veneto	Good	Good	Illegal hunting	B	
xxxx312	April 2016	Emilia Romagna	Poor	Poor	Undetermined	R, P	Sectioned **
xxxx206	October 2017	Tuscany	Good	Fair	Trauma	R, T, H,	
xxxxx310–114	November 2018	Tuscany	Good	Good	Illegal hunting	R, T	
xxxxx310–115	November 2018	Tuscany	Good	Good	Illegal hunting	R, T	
xxxxx101	January 2019	Tuscany	Good	Good	Illegal hunting	R	
xxxxxx55–1	December 2018	Tuscany	Poor	Poor	Starvation	T	
xxxxxx55–2	December 2018	Tuscany	Poor	Poor	Starvation	T	
xxxxx481	December 2018	Tuscany	Good	Poor	Starvation	T, V, H	
xxxxx806	June 2019	Tuscany	Good	Good	Trauma	R, T	
xxxxx810	July 2019	Tuscany	Good	Fair	Disease	R, T, H	
xxxxx885	June 2019	Tuscany	Poor	Good	Disease	H	
xxxxx535	December 2019	Tuscany	Good	Fair	Trauma	V, P	
xxxxx537	January 2020	Tuscany	Poor	Fair	Trauma	V, P	
xxxxx877	March 2020	Tuscany	Good	Good	Trauma	No	
xxxxx489	December 2019	Veneto	Good	Fair	Trauma	V, H	Sectioned **
xxxxx528	January 2020	Veneto	Extremely poor	Undetermined	Undetermined	V	Sectioned **
xxxxx866	August 2020	Tuscany	Good	Fair	Predation	No	
xxxxx360	August 2020	Tuscany	Good	Good	Electrocution	V, H	
xxxxx422	November 2020	Tuscany	Good	Good	Illegal hunting	R	
xxxxx036	September 2020	Liguria	Good	Fair	Illegal hunting	R, B	
xxxxx901	September 2020	Tuscany	Good	Fair	Illegal hunting	R	
xxxxx843	January 2022	Tuscany	Good	Poor	Predation	R	
xxxxx306	April 2022	Lombardy	Undetermined	Undetermined	Illegal hunting	R, H	Sectioned **
xxxxx749	July 2022	Lombardy	Poor	Good	Predation	R	
xxxxx848–1	February 2022	Tuscany	Good	Poor	Starvation	T	
xxxxx848–2	February 2022	Tuscany	Good	Poor	Trauma	No	

** previously sectioned in other facilities, B = ballistics exam, H= histological exam, P = parasitological exam, R = radiological exam, T = toxicological exam, V = virological exam, and No = no exams performed.

**Table 2:** Diagnostic tests performed and results.

	Parasitological Exam	X-Ray/Ballistics Analysis	Toxicological Exam	Virological Exam
ANIMAL TESTED	3 out of 27	14 out of 27	9 out of 27	6 out of 27
POSITIVE	2 (67%)	8 (57%)	0 (0%)	0 (0%)
NEGATIVE	1 (33%)	6 (43%)	9 (100%)	6 (100%)

## Data Availability

Not applicable.
